# A geometric framework for reaction enumeration in computational nucleic acid devices

**DOI:** 10.1098/rsif.2023.0259

**Published:** 2023-11-15

**Authors:** Sarika Kumar, Matthew R. Lakin

**Affiliations:** ^1^ Department of Computer Science, University of New Mexico, Albuquerque, NM, USA; ^2^ Department of Chemical and Biological Engineering, University of New Mexico, Albuquerque, NM, USA; ^3^ Center for Biomedical Engineering, University of New Mexico, Albuquerque, NM, USA

**Keywords:** DNA strand displacement, computational modelling, reaction enumeration, molecular geometry

## Abstract

Cascades of DNA strand displacement reactions enable the design of potentially large circuits with complex behaviour. Computational modelling of such systems is desirable to enable rapid design and analysis. In previous work, the expressive power of graph theory was used to enumerate reactions implementing strand displacement across a wide range of complex structures. However, coping with the rich variety of possible graph-based structures required enumeration rules with complicated side-conditions. This paper presents an alternative approach to tackle the problem of enumerating reactions at domain level involving complex structures by integrating with a geometric constraint solving algorithm. The rule sets from previous work are simplified by replacing side-conditions with a general check on the geometric plausibility of structures generated by the enumeration algorithm. This produces a highly general geometric framework for reaction enumeration. Here, we instantiate this framework to solve geometric constraints by a structure sampling approach in which we randomly generate sets of coordinates and check whether they satisfy all the constraints. We demonstrate this system by applying it to examples from the literature where molecular geometry plays an important role, including DNA hairpin and remote toehold reactions. This work therefore enables integration of reaction enumeration and structural modelling.

## 1. Introduction

The field of molecular programming aims to design, analyse and build information-based circuits at the nanoscale, often using nucleic acids as the underlying material and information storage medium. Toehold mediated strand displacement [1,2] has been a key mechanism used to build a variety of computational nucleic acid nanostructures and circuits using DNA [3–6] that have great potential for applications in biosensing [7,8], diagnostics [9,10] and therapeutics [11,12]. As the size and complexity of the circuits that can be constructed in the laboratory continues to increase [13], automated tools will become indispensable for carrying out the design tasks required to construct these molecular circuits, such as identifying spurious reactions that decrease the performance and scalability of such circuits [14].

A key task in molecular circuit design is that of *reaction enumeration*, that is, the problem of determining the set of reactions that could occur given a set of initial species. This can be thought of as ‘compiling’ a structural description of the circuit components into a kinetic model, which is a necessary step before analyses such as stochastic or deterministic simulations or model checking can be carried out. The resulting model is defined by a specification of the compilation process in terms of hand-crafted formal semantic rules. An early programming language for modelling strand displacement reactions was the DSD language [15], which was developed by one of us [16] and implemented in the associated Visual DSD software tool [17]. Early versions of this system were restricted to a (nevertheless quite expressive) class of linear heteropolymer structures [18] and a range of hard-coded compilation semantics [16]. Ad hoc extensions to model hairpin structures [19] and structures tethered to a surface [20] enabled data-driven simulation and modelling of localized circuits on DNA origami nanostructures [21]. To handle more complex secondary structures, such as branched or multi-junction structures, a more expressive representation for the underlying structures of the circuit components was required. Therefore, in subsequent work, a new version of DSD was developed based on a strand graph data structure [22] capable of representing arbitrary secondary structures including pseudoknots, although the compilation rules in that paper included side conditions designed to prevent pseudoknots from forming. More recently, the Logic DSD system refounded DSD on a logic programming framework that enables user-defined compilation schemes to be encoded in a PROLOG-like language that drives the reaction enumerator [23]. The Logic DSD system has been used to explore the effects of different assumptions about leak reactions on the behaviour of feedback control circuits [14]. This approach, and similar approaches [24], means that all reactions of a similar kind are either permitted or omitted from output models, without any further considerations as to their geometric plausibility, as the approach is purely syntactic in nature.

However, as the scope of experimental work in DNA nanotechnology expanded to cover more complex structures, using graph-based structure representations [22,24], the number of ‘corner cases’ tended to increase, meaning that the complexity of the rules also tended to increase to prevent unphysical reactions being returned by the algorithm while continuing to make decisions based solely on the syntax of the underlying structural representation. This has produced rule sets that can be hard for the user to understand in full detail and which may be in certain cases overly restrictive [22], preventing certain classes of reactions from being included in the model even though they might be biophysically plausible in practice. Here, we seek to address this issue via a more nuanced treatment of molecular geometry in the reaction enumeration algorithm.

Specifically, we take an alternative approach to reaction enumeration in DNA strand displacement-based molecular computing systems. Our goal is to simplify the rule set used in reaction enumeration as far as possible while also eliminating unrealistic reactions. To achieve this, we note that the purpose of the complex rule sets such as those found in previous work [22] is to produce a syntactic approximation to the set of interactions that is possible given the geometric constraints imposed by the biophysics of the DNA components that make up the system. Therefore, our approach is to integrate a geometric solver into our reaction enumeration algorithm so that much of that complexity is pushed out of the reaction enumeration rule set itself and into the constraint solving system. Furthermore, since this solver directly encodes the problem in question, rather than seeking an approximation to it via syntactic approaches, the definition of this solver can also be relatively simple to comprehend. This work thus offers a new and simple approach to reaction enumeration in DNA strand displacement systems. To our knowledge, this paper is the first work to incorporate automatic analysis of structural geometry into the reaction enumeration process.

**Figure 1.**
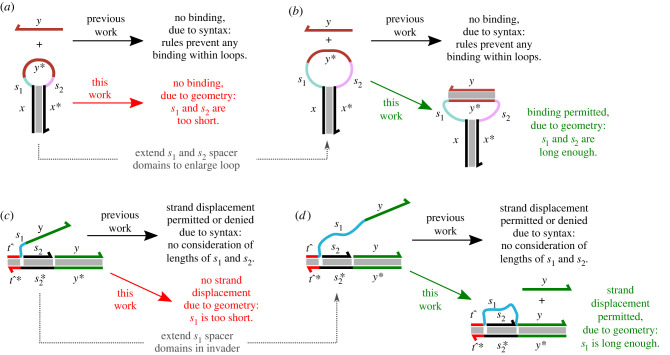
Fundamental differences between our geometric approach to reaction enumeration in DNA-based molecular systems and previous syntactic approaches, illustrated using examples from the literature. (*a*) A single-stranded oligonucleotide *y* attempts to bind with its complement y* that is located within the loop of a hairpin structure. In previous work [22,24], where entire classes of reactions are eliminated based on syntactic considerations, this reaction is never permitted because binding with a loop is never allowed. In the case here when *s*_1_ and *s*_2_ are so short that the loop contains insufficient slack to accommodate a rigid duplex formed when *y* and y* bind, the result from previous work tallies with the expected behaviour given the geometry of the system. (*b*) However, if *s*_1_ and *s*_2_ are lengthened so that the loop is large enough to accommodate the binding of *y* and y*, as shown here, the results obtained from previous work diverge from the expected behaviour given the geometry. In this work, we present a reaction enumeration system that accounts for geometric considerations such as these and can thus enumerate reactions intelligently depending on whether the biophysics of the system would actually permit those reactions to proceed. (*c*) A remote toehold strand displacement reaction [25], with a single-stranded spacer on the invader strand and a double-stranded spacer on the incumbent gate complex. In previous work, this reaction would either be permitted or denied again on syntactic considerations, without determining whether the reaction is geometrically feasible given the domain lengths. In the case when *s*_1_ is too short compared with *s*_2_, as shown here, the strand displacement reaction should not be allowed to proceed and our geometric approach will rule it out. (*d*) By contrast, if *s*_1_ is made long enough to reach across the *s*_2_ domain, as shown here, our geometric approach can detect this and include the strand displacement reaction in the enumerated reaction network.

Figure 1 illustrates this fundamental difference between the purely syntactic approach and our new approach via the example of a domain binding to its complement which is located within a loop. This will serve as a running example throughout this paper. In previous work, all such interactions would be omitted from the model based on syntactic analysis of the product structure, as such reactions would introduce a new bond within a loop and are thus forbidden on the basis that most such loops in practice tend to be too small to permit such interactions. By contrast, in our work, we carry out a geometry analysis to determine whether this is in fact the case, which may result in this reaction being included within the generated model if the loop is large enough to accommodate the bound domain. While our approach is general-purpose, in this paper, we will focus on examples that illustrate situations in which geometric considerations play a key role, such as hairpin-based examples and remote toehold strand displacement reactions [25], both of which are depicted in figure 1. Our approach is also very relevant to surface-bound localized DNA circuits [21,26], which could be explored in future work. As we shall show, our approach also allows undesired spurious binding into loops, for example, via unreacted fuel hairpins [21], to be eliminated based on similar geometrical considerations.

## 2. The strand graph structural model

Modelling and analysis are important precursors to any experimental work. This involves formally defining the syntax and semantics of the system. Here, syntax refers to precisely representing DNA structures and semantics refers to the interaction between these DNA structures in the system. In this section, we describe the ‘strand graph’ structural model of domain-level devices that we use as our basis in this work that was reported in previous work [22].

In the strand graph model, each individual DNA strand is represented by a *vertex* which we refer to by a natural number index; the set of such vertices is *V*. For convenience, we assume that the vertices are indexed sequentially, starting from zero (this is a minor departure from previous work [22], which indexed starting from one). The colour function maps each vertex *v* to a natural number index, referred to as a *colour* following previous work [22], which tracks the kind of DNA strand that vertex represents. For each vertex, there is an associated length, length(v), which is the number of domains present in the strand: each of these is represented as a ‘site’ on the vertex in the strand graph model. These sites are ordered as per the sequence of domains in the strand, moving from the 5’ end of the strand to the 3’ end. Thus, each domain in the system is represented as a pair (*v*, *s*) where *v* is the index of the vertex corresponding to the strand it is on and *s* is the index of the corresponding site within that vertex, such that 0≤s<length(v); the set of such sites is referred to in [22] as the set of *legal* sites for the strand graph.

Domains can be free or bound and may either be short *toehold* domains tˆ (typically having a length of 5–8 nucleotides) or *long* domains. We assume that nucleotide sequences are assigned so that each domain *d* will only bind to the complementary domain d*. Bonds between domains are modelled by edges between sites: the set *A* in the strand graph is the set of all possible edges that could be formed between complementary domains. These edges are known as the *admissible* edges and are stored as pairs of (vertex,site) pairs. The set *E* records the set of *current* edges, that is, the subset of admissible edges that are actually formed in the current state of the strand graph. It is assumed that toehold domain bindings are not strong, so they can spontaneously bind and unbind, but long domains bind irreversibly. The toehold(a) predicate defined in the strand graph maps every admissible edge to True if that edge represents a bond between toehold domains and False otherwise. Thus, the full definition of a strand graph *G* consists of the elements G=(V,length,colour,A,toehold,E), as outlined above. The formal definition after Petersen *et al.* [22], specifically, definition 4 of that paper, is reproduced in the electronic supplementary material, for reference.

**Figure 2.**
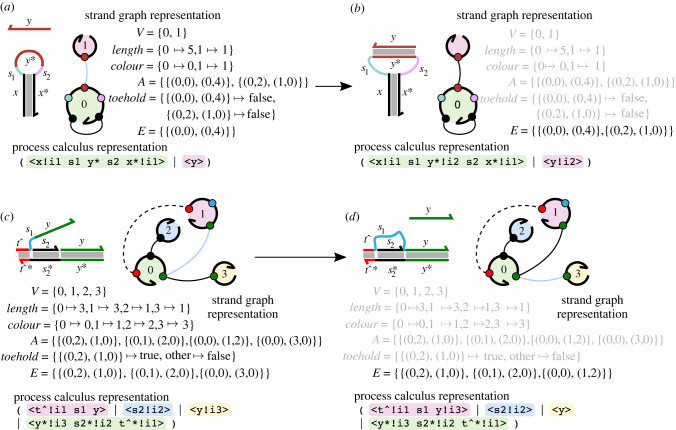
Strand graph examples. (*a*) The initial state of the hairpin binding example from figure 1*a*,*b*, with the complementary single strand unbound from the hairpin, rendered as a strand graph. The visualization of the strand graph, styled after previous work [22], with the nodes representing the vertices of the strand graph and the small discs on the vertices representing sites, numbered in order from 5’ to 3’. The individual sites are coloured to correspond to the colours of the domains in the graphical representation of the system. The number on the node represents its index from the set *V*. The shading colours within the nodes represent their colours, colour(V). Solid lines between sites represent admissible edges between non-toehold domains while broken lines between sites represent admissible edges between toehold domains. Light-coloured edges are those that are admissible but the corresponding bonds are not actually formed (i.e. in *A* but not in *E*); dark-coloured edges represent bonds that are actually formed (i.e. members of *E*). The visualization is accompanied by a formal statement of the components of a corresponding strand graph G=(V,length,colour,A,toehold,E). Following previous work [22], a more convenient text-based ‘process calculus’ representation of the strand graph is included below the strand graph representation here and below, with strand colours represented as highlighting behind the process calculus syntax. (*b*) Strand graph example corresponding to the post-reaction bound state of the hairpin-binding system, as illustrated in figure 1*b*. In this case, the only change is the formation of the bond between the *y* and y* domains. The structure of the strand graph remains largely the same (unchanged parts of the definition are rendered in light grey); the only thing that changes is the set of current bonds *E* which sees the additional bond added. (*c*) Strand graph rendering of the pre-reaction state of the remote toehold strand displacement example from figure 1*c*,*d*, following the graphical and notational conventions outlined above. (*d*) Strand graph rendering of the post-reaction state of the remote toehold example shown in figure 1*d*.

Strand graph representations for the structures introduced in figure 1 are presented in figure 2 to illustrate the encoding, depicting the initial and final states for each example. Transformations of the strand graphs corresponding to reactions are modelled by simply transforming the edges between the vertices: edges are removed from *E* to represent breaking bonds between domains and added to represent the formation of new bonds between complementary domains. As we shall see below, these transformations can be specified using reduction rules that change the form of the strand graph. The examples in figure 2 also show a representation of each strand graph in the so-called ‘process calculus syntax’ introduced in [22]. This is a text-based representation of the strand graph where each strand is presented as an ordered sequence of domains within angle-brackets, with the formed bonds given names and preceded by an exclamation mark. The notation *d*!*i* thus refers to domain *d* which forms one side of the bond named *i*; for well-formedness the other side of that bond must thus be d*!*i* and the bond name *i* can only appear twice. For example, in figure 2*a*, the hairpin strand is represented by the process calculus syntax 〈*x*!*i*_1_ *s*_1_ y* *s*_2_ x*!*i*_1_〉, with the bond *i*_1_ representing the stem of the hairpin. Here and henceforth, we will use the process calculus syntax as a convenient means to represent strand graphs in text and figures.

We note that, in our implementation, we also store the length of each domain in nucleotides; this was not necessary in the previous work but here we will need it to calculate the biophysical constraints imposed by the lengths of domains and the bonds that are formed within the structure. In addition, to rule out the unphysical corner case of a double helix that can form a ‘hairpin’ with a loop length of zero nucleotides, we statically rule out any system in which a domain and its complement are immediately adjacent on a strand: there must be some other domain (having non-zero length) in between. In the strand graph parlance, this means that there can be no admissible edge of the form {(*v*, *n*), (*v*, *n* + 1)} between two *directly adjacent* sites on any vertex.

## 3. A general-purpose framework for geometric reaction enumeration

**Figure 3.**
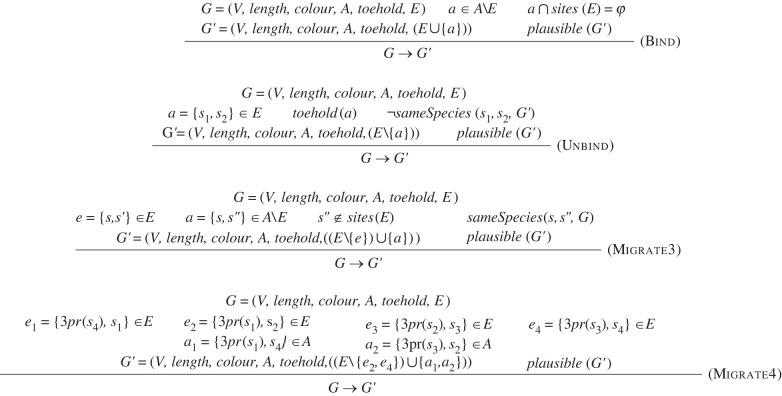
Inference rules defining a geometrically aware transition relation (→) between strand graphs.

Semantic rules allow the automatic computation of interactions between strands which are applied to a collection of DNA nanostructures in a given context. To this end, our first step is to define our reaction enumerator at a high level. Our goal here is to show how the use of a geometric predicate can greatly simplify the complexity of semantic rules for deriving physically realistic chemical reaction networks from syntactic descriptions of DNA strand displacement systems. As such, we do not commit ourselves to any particular biophysical model of molecular geometry nor to any specific algorithm for solving constraints over structures. We therefore present a minimal rule set which can be specialized to particular choices of biophysical model and constraint solving system; we present one such specialization below. The general-purpose rules are presented in figure 3.

The goal of these rules is to simplify the enumerator definition (and implementation) by removing the complex side-condition predicates that produce many of the corner cases that make strand graph systems hard to reason about, e.g. the hidden and anchored side conditions from our previous work [22]. The only external predicates required by the definition in figure 3 are sameSpecies and  plausible. As mentioned above,  plausible could be defined in a number of different ways; therefore, the set of rules from figure 3 defines a general *framework* for implementing geometrically aware algorithms for reaction enumeration.

A ‘species’ in a strand graph is defined as a connected component of the graph; the predicate $sameSpecies(s,s^{\prime },G)$ returns True if the two sites *s* and *s*′ occur in the same connected component of the strand graph *G* and False otherwise. This predicate can be implemented via strand graph traversal. In addition, the 3pr(s) function returns the domain located directly adjacent to domain *s* in the 3’ direction, if it exists.

The ‘Bind’ rule requires that the new edge being formed (*a*) is a legal edge that could be formed (i.e. in the set *A* of *admissible* edges) but not already formed (i.e. not in the set *E* of *current* edges). In addition, neither of the sites involved should already be bound to anything else, which is enforced by the requirement that a∩sites(E)=∅, which says that none of the sites involved in *a* are part of an edge from the set *E* of *current* edges. The ‘Unbind’ rule requires that the edge being removed (*a*) must be a toehold edge. In addition, spontaneous unbinding of toeholds within a species is prevented by the requirement that the two sites *s*_1_ and *s*_2_ initially joined by *a* must be in different species in the new strand graph *G*′. The ‘Migrate3’ rule allows site *s*′ to be replaced by *s*″ in being bound to *s*, provided that *s* and *s*″ are in the same species in the initial graph *G* and provided that *s*″ is not already involved in any other bonds. The new edge *a* must be admissible but cannot already be formed (i.e. in *A* but not in *E*). Finally, the ‘Migrate4’ rule allows four bonds *directly* around a four-way junction to swap binding partners in a four-way migration reaction, removing the bonds {*e*_2_, *e*_4_} and replacing them with {*a*_1_, *a*_2_}. The two new bonds must be admissible, (i.e. in *A*); this enforces the requirement that the domains around the junction must be of the correct domains and complementarities to be able to swap binding partners in this manner. *Importantly, all of these reaction rules are predicated on the product species all being physically plausible as per our geometric constraint satisfiability checking algorithm of choice.*

In our previous work [22], similarly named rewriting rules were defined. To specify these rules, however, auxiliary predicates were defined that examine the surrounding context in the strand graph structure to serve as side-conditions limiting whether that rule could be applied. The two key auxiliary predicates were hidden and anchored: the predicate hidden(e,E) is True if one end of the new edge *e* would be contained within a closed loop given the set *E* of existing bonds; this prevents binding into a hairpin. The predicate anchored(e,E) is True if edge *e* connects sites that are held closely together, either by directly adjacent bound domains or by an *n*-way junction. This prevents spontaneous unbinding of toeholds within a structure and also requires an invader strand to bind via a nucleation site for strand displacement to take place. If these conditions are satisfied, then the rule can be applied to infer a strand graph transition. Importantly, these auxiliary predicates were based on matching the context and required graph traversal to determine whether the rules may be applied. As the complexity of the structure increases, these auxiliary functions become expensive to compute; they are also complex to state and understand. In this work, we remove these auxiliary functions and introduce the geometric features from our work on localized circuits to determine whether a given reaction is plausible or not.

We adopt a similar chemical reaction semantics to that used in our previous work [22] to convert these transitions between strand graphs into either unimolecular or bimolecular chemical reactions in a compiled chemical reaction network (CRN). As the technical contribution of this paper is in the derivation of the individual transitions based on geometric considerations, we re-use the CRN semantics from that paper directly and thus do not reproduce those definitions here; we refer the interested reader to section 3.4 of Petersen *et al.* [22] for the details. In addition, following the approach of that paper, here we also consider the merging of reactions based on separation of time scales to fall outside the scope of our immediate technical contribution. This is an important topic, which has been dealt with in existing tools such as Peppercorn [24] and the DSD system [16,23], whose approaches to merging are separate from the enumeration of the underlying state transitions and reactions themselves. As reaction enumeration is our central object of interest, we do not merge reactions here, although there is no reason why a reaction enumeration approach such as ours could not be combined with a separate reaction merging algorithm.

## 4. A structure sampling approach to geometric reaction enumeration

The logical rules from figure 3 define a highly general framework for enumerating reactions with a geometric component. Implementing this framework essentially reduces to providing an implementation of the  plausible predicate over strand graphs, which tests whether a given product strand graph is geometrically plausible and thus whether a candidate reaction can proceed when geometric considerations are incorporated into the reaction enumerator. While there are a number of different ways these constraints might be solved, here, we adopt a straightforward probabilistic approach based on generating candidate physical structures for the strand graph at random using a simplified biophysical model. This approach, outlined in this section, builds upon and generalizes our previous work on structure sampling that was restricted to simple linear chain structures [27]. We first outline the assumptions in our biophysical model before describing the data structures and transformations that we use to define the sampling procedure, which produces an approximate decision procedure for this constraint problem.

### 4.1. Biophysical model

The mechanical properties of DNA, such as stiffness or flexibility, play an important role in the biophysics of DNA nanostructures; the stiffness of double-stranded DNA provides the basic shape of a DNA structure and the flexibility of single-stranded DNA allows any joints to move freely. Here, we adopt a very simple model of the biophysics of DNA, inherited from our previous work [28].

We assume that double-stranded domains behave as rigid rods of fixed length, as they are assumed to be much shorter than the persistence length of double-stranded DNA. We assume that single-stranded domains are flexible freely jointed chains and may thus be oriented in any direction and take on any length between zero and their maximum possible length value. We also disregard the width of the DNA duplex in our geometric model, so that the 5’ end of one strand in a duplex is located at the same coordinates as the 3’ end of the other strand. We assume that nicks between double-stranded domains are infinitely flexible.

To solve the constraints required for our reaction enumerator, knowledge of the physical lengths of domains is required to determine the fixed or maximum distance between different parts of the DNA structure. This physical distance is dependent on the number of nucleotides in the domain and the physical length per nucleotide. We assume a function domainLength that maps each domain (and thus site in the strand graph) to the number of nucleotides in the corresponding domain sequence. (Note that we do not consider sequence effects in our model.) To convert into length units, we assume the physical length per nucleotide to be 0.68 nm for a single-stranded and 0.34 nm for double-stranded DNA; the values were taken from the literature [19,25,28].

The choices we have made here will affect the results from our model and will also impact our ability to easily solve the underlying constraints: a more complex model would require greater computational resources to solve, with the ultimate limit being all-atom simulations of the underlying DNA nanostructures. We anticipate that these simplifications may impact results quantitatively at the margins but should not qualitatively affect the predicted behaviour of the system. Notably, we neglect the width of the DNA double helix, which is around 2 nm in reality. This may mean that hairpin loops would actually be slightly more permissive to binding than we predict here, as the additional width of the double helix would mean that a loop of given size would not need to be quite so tightly curved to close the loop successfully. Since we are neglecting the width of the double helix, it also makes sense to ignore helical twist, as the additional distance a strand would need to reach ‘across’ the double helix to bind with a complementary strand would only be a nanometre or so. Given that domain-level CRN models of DNA systems make a number of other simplifying assumptions, including the all-or-nothing interaction behaviour of domains, we believe that any inaccuracies caused by our simplified biophysical model will be minor.

### 4.2. A data structure for structure sampling

**Figure 4.**
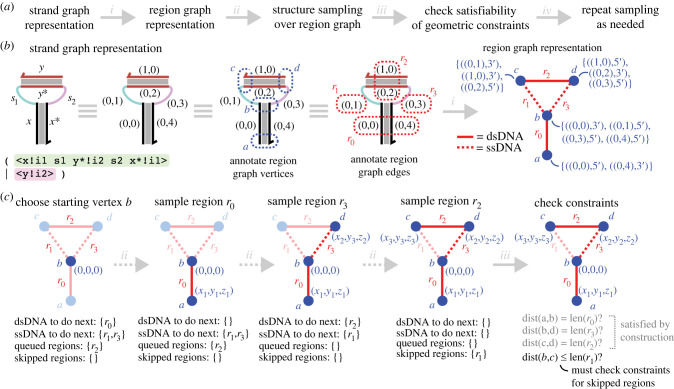
Illustration of structure sampling over region graphs. (*a*) Overall workflow for our probabilistic approach to geometric constraint solving. (*b*) Detail of the translation from strand graphs into the ‘region graph’ representation for the bound state of the hairpin-binding example from figure 2*b*. We show the diagram annotated first with the corresponding vertex and site indexes, then with the associated vertices and edges of the region graph annotated, before showing the region graph itself. The vertices of the region graph are labelled *a*, *b*, *c*, *d* and the regions themselves are labelled *r*_1_, *r*_2_, *r*_3_, *r*_4_. The vertices of the region graph are labelled with the strand graph sites and associated ends (5’ or 3’) that are grouped together into that vertex. (*c*) Example of structure sampling and constraint checking for the hairpin-binding example. The algorithm is detailed in the text, but in essence we start at a maximum-degree vertex, set that to be the origin, and sample coordinates at a randomly chosen neighbouring vertex connected via dsDNA regions preferentially before sampling those connected by ssDNA regions. We stop when all vertices have coordinates sampled for them. The final step is to check the associated constraints given the sampled coordinates; the constraints corresponding to the regions that were explicitly sampling should be implicitly satisfied, in theory requiring only the constraints corresponding to any skipped regions (those that were not directly sampled) to be checked explicitly. In the case of this hairpin-binding example, this checks whether the regions *r*_3_ and *r*_2_ in the graph were sampled so that vertex *c* ends up close enough to vertex *b* such that the length constraint on region *r*_1_ is not violated.

The first step towards implementing our structure sampling approach to geometric constraint satisfaction is to define a suitable data structure over which to sample. The strand graph data structure is well suited to domain-level reaction enumeration because it uses sites to represent domains directly. However, for structure sampling, we are primarily interested in the coordinates not of the domains themselves but of the boundaries between regions of either single- or double-stranded DNA, as these are where the structure may bend or flex as required to satisfy the geometric constraints. Thus, here we outline the conversion of strand graphs into *region graphs*, an intermediate data structure that we will use to define the set of geometric constraints arising from a particular strand graph; this overall workflow is summarized in figure 4*a*. The key idea behind the region graph is to convert into a graph structure where the vertices represent the locations for which we need to find satisfying coordinates, with the edges representing the connections between these, which could represent regions of either single- or double-stranded DNA.

In our previous work [28], we defined a notion of *condensing* a process calculus representation of a structure by collapsing adjacent single-stranded or double-stranded domains so that the domain boundaries became the only important positions represented in the constraint problem. In the context of strand graphs, however, this is more of a challenge to implement because combining two domains into one requires potentially a large chunk of the entire strand graph structure to be relabelled to maintain consistency. Therefore, here we propose an alternative definition (equivalent to the one from our previous work [28]), in which we instead assign domains to *regions* that are then used as the basis for defining the constraint problem. This approach avoids the need to relabel the strand graph.

We will illustrate the conversion of strand graphs to region graphs through an example: figure 4*b* presents an overview of this conversion for the example of a single-strand binding within a hairpin loop that was introduced in figure 1*b*. In this example, the structure is first shown with an example labelling of (vertex,site) pairs, as per the strand graph definition. Then, the parts of the structure that correspond to the vertices in the region graph representation are shown outlined and labelled in blue. Intuitively, these correspond to the termini (either 5’ or 3’) of the domains at either end of a contiguous run of double-stranded or single-stranded domains, grouped together with any additional domain termini that are either directly adjacent on the same strand or else directly opposite at the end of a duplex. The strand graph sites corresponding to each edge of the region graph are highlighted in red; due to the nature of this example, each region consists of a single-stranded domain or single pair of bound double-stranded domains. We then use these vertices and edges to create an undirected graph, as shown on the right-hand side of figure 4*b*. For an input strand graph *G*, we write regiongraph(G) for the associated region graph; this mapping is defined formally in the electronic supplementary material. This conversion can be implemented via a straightforward partitioning algorithm whereby we form region graph vertices that each contain a single domain terminus from the strand graph and repeatedly combine parts of the partition containing domain termini that should occur together in the same vertex in the final region graph.

### 4.3. Forming geometric constraint sets from strand graphs

We previously reported a geometric constraint solving approach for tethered molecular devices [28]. Here, we use a similar approach, whereby each structure is converted into a set of geometric constraints on the coordinates of different parts of that structure. If this set of constraints is satisfiable—that is, if the constraints can all be satisfied simultaneously by some assignment of coordinates to the variables—then the structure *could* form based on geometric considerations.

The first step towards forming the set of geometric constraints associated with a strand graph *G* is to form the associated region graph, regiongraph(G)=(RGV,RGE), as outlined above and defined formally in the electronic supplementary material. This process implicitly implements the ‘hybridization constraints’ that we used in the previous work [28], as domains hybridized to each other end up being collapsed into the same vertices of the region graph (which is acceptable because we neglect the width of the DNA double helix in our simplified biophysical model). Thus, the only constraints that remain to be solved are distance constraints between the nodes of the region graph. These may take two different forms:

— $dist(N,N^{\prime })=d$, which specifies that the distance between nodes *N* and *N*′ must be precisely equal to *d* (suitable for double-stranded regions), and
— $dist(N,N^{\prime })\leq d$, which specifies that the distance between nodes *N* and *N*′ must be at most *d* (suitable for single-stranded regions).

We assume that the input strand graph *G* has been converted into the corresponding region graph, regiongraph(G)=(RGV,RGE). To decide which kind of constraint to use for a given edge of a region graph, we write dsedges(RGE) for the subset of edges from the region graph that corresponds to *double-stranded* regions and ssedges(RGE) for the subset of edges from the region graph that corresponds to *single-stranded* regions. We write *edgelength*(*e*) for the length in nanometres associated with the region graph edge *e*, which corresponds to the *total* length for double-stranded domains and the *maximum* length for single-stranded domains. Then, we can define the set of constraints, C(G), that corresponds to the strand graph *G*, as follows:

$$\begin{array}{rl} C(G) & =\{dist(N,N^{\prime })=d|\{N,N^{\prime }\}\in dsedges(RGE)\\ & \;\;\wedge \;edgelength(\left\{N,N^{\prime }\right\})=d\}\;\cup \\ & \;\;\{dist(N,N^{\prime })\leq d|\{N,N^{\prime }\}\in ssedges(RGE)\\ & \;\;\wedge \;edgelength(\left\{N,N^{\prime }\right\})=d\} \end{array}$$

The strand graph structure *G* is *plausible*, which we write as  plausible(G), iff there exists a mapping of coordinates to the region graph vertices such that all of the constraints in C(G) are satisfied simultaneously.

### 4.4. A structure sampling approach to geometric constraint solving

Having formed the region graph regiongraph(G) and constraint set C(G), as outlined above, the second step is to attempt to find candidate coordinates for the vertices in the region graph (that is, the junctions between regions) that satisfy all of the constraints in C(G). We now describe an algorithm that generalizes our previous work [27] to use structure sampling over region graphs as a probabilistic method to approximately decide constraint satisfiability checking over arbitrary connected strand graphs. This simple approach generates random structures and tests them to see whether they actually satisfy the constraints.

When generating such sampled structures, our goal is to maximize the probability of finding a satisfying sample in the allotted number of attempts, if one exists. We pick a starting vertex that we call the origin and sample directions and lengths for regions one at a time, always adding incrementally to the existing structure. We sample the direction of each region from a uniform distribution of angles in three-dimensional space by choosing polar and azimuthal angles from a uniform distribution over the interval [0, 2*π*) and correcting the polar angles as outlined in our previous work [27]. The lengths of double-stranded DNA regions are fixed. Single-stranded DNA regions are assumed to be highly flexible and we randomly sampled the lengths of the single-stranded regions from a worm-like chain distribution [29] over lengths between 0 nm and the maximum possible extension of the domain in nanometres. This sampling process could be done in a number of different ways, all of which must essentially fall back on heuristics to pick the starting point for sampling and the order in which to sample new coordinates.

Our chosen sampling heuristic is outlined in figure 4*c* and summarized in pseudocode form in algorithm 1. This heuristic is based on the observation that we want to sample single-stranded domains as late as possible because they provide the ‘slack’ that enables the regions to adopt a satisfying conformation if there is any possibility of constraints being unsatisfiable, which only arises when a loop forms in the structure. It is worth noting that the samples we generate are only *candidate* structures: in a cycle some vertices will be constrained from both sides and thus we sample one side and check the resulting constraint for the other side. By deferring the sampling of single-stranded domains until later, where possible, we maximize the likelihood that the final constraint that is only checked is one corresponding to a single-stranded region. This means it only needs to fall between distance zero and the maximum possible length for that region of the coordinate already sampled for the other end of the region, as illustrated in figure 4*c*. Conversely, if the final region were double-stranded, whose length is fixed, then the probability of sampling a coordinate at precisely the right distance from the vertex at the other end of the region would typically be vanishingly small.


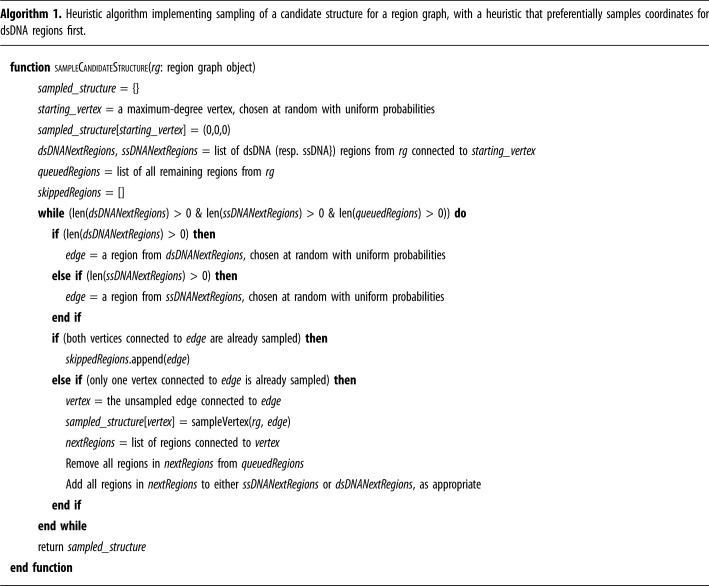


Having generated a candidate structure, we next check the coordinates against the constraints implied by the region graph to see if that sampled structure satisfies the constraints. Note that, in forming the region graph, we have already effectively ‘substituted away’ the equality constraints in the former ‘hybridization constraints’, leaving only distance constraints. These distance constraints are fully specified by the information in the region graph, namely, the type of each edge (ssDNA or dsDNA) and the corresponding length in nanometres (which is a maximum length in the ssDNA case and a fixed length in the dsDNA case). The simplest approach here is to simply loop over every edge in the region graph, obtain the sampled coordinates for the two ends, and check that they are all satisfied. However, as outlined in figure 4*c*, the structure sampling process ensures that the constraints are satisfied by construction for all but those regions which are not explicitly sampled (i.e. the final regions that serve to close a loop), meaning that these final constraints are the only ones that really need to be explicitly checked in practice.

The sampling procedure outlined above can be used to approximate constraint satisfiability, via the following algorithm which is parametrized by a positive integer *n* representing the number of sampling attempts to make before giving up. The algorithm is presented as algorithm 2 but its core is very simple: repeat the above process *n* times; when a structure sampling is found that satisfies the constraints, return True. If no such sampling is found after *n* attempts, return False.


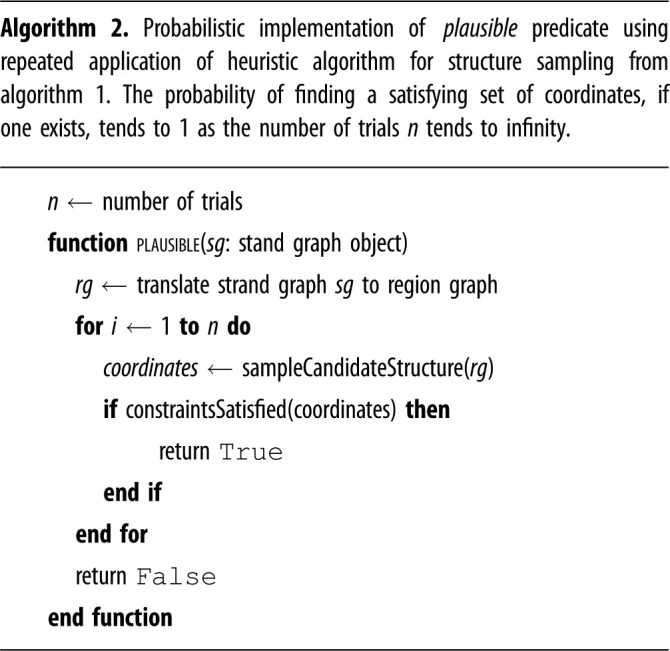


From a qualitative standpoint, the parameter *n* can be tuned to trade off the accuracy of the approximation to true constraint satisfiability against the length of time required. In particular, larger values of *n* will be more likely to find structures that are technically plausible but rare due to only occurring in a small number of plausible configurations, but will take longer to reject completely implausible structures. On the other hand, smaller values of *n* will run faster in more normal cases but are more likely to reject some plausible structures. It is worth noting that, when paired with a reaction enumeration algorithm, this approach is somewhat robust to reactions that are theoretically possible but relatively unlikely to occur. This is because the number of satisfying structures may be viewed as a proxy for how likely the molecules are to find a valid structure, when they are the reactants of a candidate reaction. If the number of solutions is small then the molecules would be relatively unlikely to adopt such conformations and thus the reaction should be unlikely to occur. In general, reaction enumeration algorithms do not consider the relative likelihoods of different reactions, meaning that low-probability reactions with low reaction rates, such as leak reactions, can ‘pollute’ the resulting CRN with large numbers of reactions that may occur infrequently in an actual simulation. In the sampling approach, the corresponding product structures will be less likely to return a satisfying structure sample in *n* tries and will therefore be less likely to be included. If these reactions are not likely to occur, the effect on the results of a simulation is likely to be small, though it is possible that additional high-priority reactions could be enabled by an initial low-probability event. We note also that all domain-level models generated by reaction enumeration algorithms are simplified models of the true dynamics of the structure, and yet these models can still be of great practical utility. Finally, the parameter *n* also places an upper bound on the running time of the algorithm, which is another desirable property for integration into the inner loop of a reaction enumerator.

## 5. Prototype implementation

To test our approach, we implemented our structure sampling algorithm in a Python-based prototype geometric reaction enumerator based on a strand graph representation of DNA nanostructures. Our reaction enumerator uses the semantic rules specified in figure 3 to define the possible transitions. Our implementation converts the strand graph into a region graph using a simple algorithm that iterates across the sites in the strand graph, adding them to a data structure representing the region graph iteratively merging the nodes of this graph to produce the final region graph that is then used for structure sampling. We refer the reader to the electronic supplementary material for a formal definition of the region graph and pseudocode outlines of the key algorithms associated with the region mapping and the novel components of our reaction enumeration algorithm. Open source code for our prototype implementation is available online.^1^

## 6. Examples

We tested our system by enumerating reaction networks for several examples from the literature, with a focus on systems where geometric considerations are important. First, we studied the simple example of a strand binding inside a loop, introduced in figure 1. We then consider several remote toehold reactions [25], also introduced in figure 1. These results show that incorporating our geometric solving approach can enumerate the reactions in these systems such that certain reactions are included or omitted based on geometric considerations, as one would expect. All tests were carried out on a 2017 Apple MacBook Pro with a 2.3 GHz Intel Core i5 processor and 8 GB of RAM. Example output from our prototype implementation is included as figures S1–S6 in the electronic supplementary material.

### 6.1. Binding within a hairpin loop

**Figure 5.**
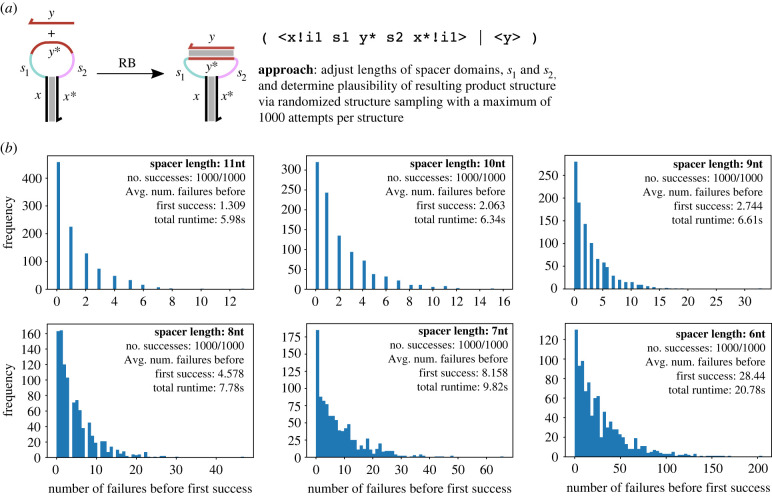
Illustration of the randomized structure sampling approach to structure plausibility checking for the example of a strand binding into a hairpin loop. (*a*) Cartoon of the possible binding reaction from this running example system, also shown is the process calculus representation of this system. The single-stranded domain 〈*y*〉 may bind to its complement y*, provided that the spacer domains *s*_1_ and *s*_2_ are long enough to accommodate the rigid double helix that would be formed. (*b*) Histograms showing the frequency distribution of the number of structure trials required to find a satisfying set of coordinates for the region graph, following a process similar to that outlined in figure 4*c*. Distributions are shown for lengths of *s*_1_ and *s*_2_ ranging from 6 to 11 nucleotides each; for 5 nt and below, no satisfying coordinate sets could be found within the allotted 1000 attempts per structure. We take this to mean that any satisfying structure is highly physically unrealistic or none exists, meaning that geometry forbids the binding reaction in these cases. With shorter spacers we require more attempts on average to find a solution, which is to be expected given that these structures are closer to the point at which the product structure becomes implausible. Importantly, the line between plausible and implausible structures is very clear in this case, with an average of 28.44 attempts required to find a plausible structure required in the 6nt spacer case, but no solution found within 1000 attempts in the cases for 5 nt and below. This suggests that our probabilistic approach can approximate geometric constraint satisfiability. The total run time for 1000 attempts is shown in each case; for 5 nt spacers the total run time was 641.67s. Further run-time analysis is presented in figure S7 in the electronic supplementary material.

To test the geometric features of our system, we first designed a simple example where a domain binds to its complement within a hairpin loop. We have used this system as a running example in figures 1, 2 and 4. Here, we note that whether or not the 〈*y*〉 strand can successfully bind to its complementary domain 〈y*〉 in the hairpin loop is a function of the domain lengths, including not just the length of the *y* domain itself but also the lengths of the spacer domains *s*_1_ and *s*_2_. Here, we aimed to show that the different cases can be distinguished by our geometric reaction enumerator by providing different combinations of the lengths of the *y*, *s*_1_ and *s*_2_ domains and using our geometric solver to check if the product structure is feasible or not, using structure sampling. That is, the system checked whether the reaction illustrated in figure 5*a* can occur or not. For simplicity of conducting experiments and displaying the results, the lengths of the *s*_1_ and *s*_2_ domain were varied together in each case. The lengths of the *x* and *y* domains were not changed so that we could analyse the effect of varying the lengths of the spacer domains *s*_1_ and *s*_2_.

The results from these tests are summarized in figure 5*b*, which uses histograms to illustrate the number of sampling trials required to find a plausible configuration for the product structure for various lengths of the spacer domains (*s*_1_ and *s*_2_) in nucleotides. In these tests, the lengths of the *x* and *y* domains were both 20 nucleotides and the lengths of *s*_1_ and *s*_2_ domains were varied starting from 11 nucleotides and counting down to 0 nucleotides (i.e. no spacer domain at all). Our goal was to gain some insight into the probabilistic behaviour of our randomized structure sampling algorithm. Therefore, we carried out 1000 structure sampling tests in each case and counted the number of successes and failures, as well as the number of sampling trials required to find a plausible structure in the successful runs. Failure was defined as failure to find any satisfying coordinates for the resulting structure in 1000 attempts.

When the spacer domains were long enough, the binding reaction was always possible. The longer the domains, fewer sampling trials were required to find a solution; this is reported as the average number of failures before a solution was found. This is as expected given that longer domains have more ‘slack’ in the structure, which means that there is likely to be a larger set of satisfying coordinates from which to choose. As the spacers shorten and approach the point at which the structure becomes implausible, the number of trials required increases as the solution space shrinks. Eventually, when the length of spacer domains becomes too short, the sampling algorithm was unable to find any satisfying solution in the allotted 1000 attempts, meaning that our algorithm decides that the binding reaction was not possible. In this particular scenario, when the spacer domain lengths were each 5 nucleotides or shorter, for a total of 10 nt for the two spacer domains (with the domain length of *y* fixed at 20 nt), the solver could not find any solution. This factor of two derives from the difference in the assumed lengths per nucleotide between single-stranded and double-stranded DNA, as outlined above. In all cases, a solution was either found in all 1000 trials or not found at all, that is, there were no structures for which a plausible structure was found in some runs but not all. This is a promising result as the clear and sharp dividing line between the plausible and implausible structures as determined via our structure sampling approach suggests that it provides a good approximation to the underlying notion of constraint satisfiability.

(We note that there is actually a solution to the constraint problem for the 5 nt spacer case, where the double-stranded domain is exactly the same length as the spacers, producing the highly unrealistic structure in which the double-stranded domain is exactly parallel to the two spacers and essentially lies on top of them to complete the loop. This requires the angles between the spacer domains and the double-stranded domain to be precisely 180°. Sampling a single real number from the corresponding interval in this manner has probability zero, therefore, it is not a significant problem that our sampling procedure fails to find such solutions, which are already at the very limit of physical plausibility.)

The total run time is also presented in figure 5*b* for each set of 1000 attempts. We note that, for the satisfiable structures, the easier it is to find a solution, the shorter the run time. For a case where no satisfying samples can be found, such as the 5 nt spacer case, the total run time was much longer as the solver had to attempt 1000 failed samplings 1000 times before giving up and declaring the constraints unsatisfiable. We note that the run time quoted in figure 5*b* for the 5 nt spacer case (641.67 s) is for 1000 repeated attempts, so in an actual reaction enumeration application the actual time taken to decide that this structure is implausible would be just 0.641 s. Further analysis of the run time of the successful examples from above is presented in figure S7 in the electronic supplementary material.

The time required for our system to determine that a structure is unsatisfiable could be reduced by setting a smaller number of attempts than 1000 or by re-implementing our prototype in a language more performant than Python. Reducing the number of attempts should improve total run time, by attempting fewer samplings overall, at the cost of increasing the probability of missing some satisfying solutions that might have been found if more samplings had been attempted. This would largely be an issue for more highly constrained systems where there are relatively few satisfying structures.

### 6.2. Remote toehold reactions

**Figure 6.**
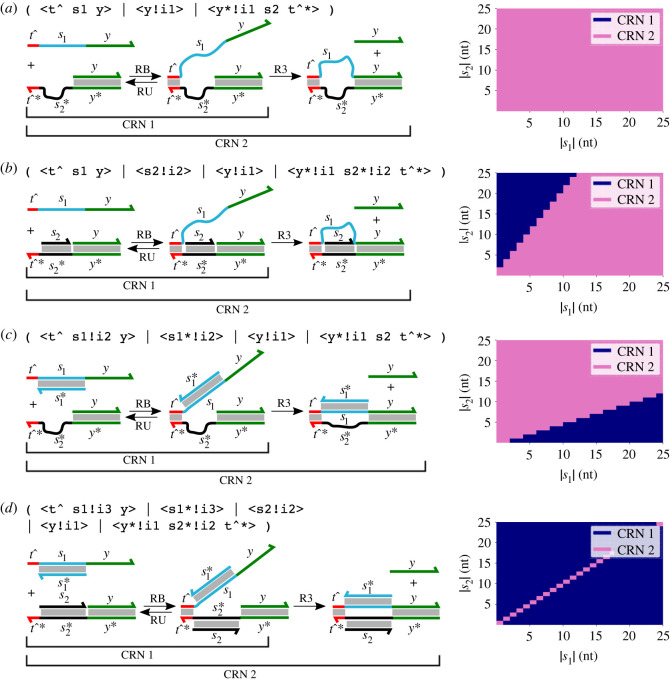
Results of applying geometric reaction enumeration by structure sampling to remote toehold reactions [25]. On the left-hand side of each panel, the process calculus representation of the input is presented along with a diagram representing the possible reactions. In each case, we use CRN 1 to refer to the toehold binding and unbinding reactions, which should be possible in all cases. Depending on geometry, a strand displacement reaction may also occur; we define CRN 2 as including this reaction also. The lengths of the spacers *s*_1_ and *s*_2_ and the nature of the spacer domains (single-stranded or double-stranded) determines whether the strand displacement reaction is possible, and thus whether CRN 1 or CRN 2 is enumerated in each case. The right-hand side of each panel contains a heatmap showing which CRN is enumerated for all combinations of spacer lengths (written as |*s*_1_| and |*s*_2_|) between 0 and 25 nucleotides. In total, 1000 attempts at structure sampling were carried out in all cases. (*a*) Both spacers are single-stranded. In this case, CRN 2 is enumerated in all cases due to the flexibility of the spacer domains. (*b*) Spacer *s*_1_ on the invading strand is single-stranded whereas spacer *s*_2_ on the gate complex is double-stranded. In this case, CRN 2 is enumerated whenever *s*_1_ is long enough to reach past *s*_2_ to access the displacement domain *y*. The slope of the transition between CRN 1 and CRN 2 regions is determined by the ratio of the lengths assumed for nucleotides in single-stranded and double-stranded domains. (*c*) Spacer *s*_1_ on the invading strand is double-stranded whereas spacer *s*_2_ on the gate complex is single-stranded. In this case, CRN 2 is enumerated whenever *s*_2_ is long enough to reach past *s*_1_; this is the dual of the previous case. (*d*) Both spacers are double-stranded. In this case, CRN 2 is enumerated only when both spacers are precisely the same length, because we allow no flexibility at all in the lengths of double-stranded domains, which are modelled as rigid rods.

Molecular geometry can also affect strand displacement reactions in the sense that geometry may prohibit or allow the reaction to occur, in particular, in the case of remote toehold reactions. In a remote toehold reaction, a spacer is introduced between toehold and displacement domain on the invader strand and/or incumbent gate complex, and it has been experimentally demonstrated that modulating the nature of these spacers affects the strand displacement reaction [25]. Here, we show that our geometric solver can automatically determine whether or not branch migration and strand displacement can occur, depending on the kinds and lengths of spacer domains involved. Specifically, in the examples from figure 6, we seek to determine whether strand displacement across the 〈*y*〉 domain occurs, depending on the relative lengths of distinct spacers on the invading and substrate strand (*s*_1_ and *s*_2_) domains and whether the spacer domains are single- or double-stranded. These results determine whether the enumerated chemical reaction network (CRN) contains just the toehold binding and unbinding reactions or the additional step of strand displacement via three-way branch migration.

Figure 6 presents the results of reaction enumeration for remote toehold systems when the length and nature of each of the spacer domains is modulated. The four panels enumerate the possible choices for the nature of the spacers on the gate complex or the invader strand, which may both be either single-stranded or double-stranded. The lengths of the spacers were varied from 0 nt to 25 nt. In each panel, the CRN representation of the reaction is shown on the left-hand side and a heatmap illustrating the reaction enumeration results is shown on the right-hand side. We define ‘CRN 1’ as the toehold binding and unbinding reactions only and ‘CRN 2’ as containing those reactions plus the subsequent strand displacement reaction. In the heatmaps, the combinations of spacer lengths for which only CRN 1 was found to be feasible correspond to the blue region, whereas the pink region corresponds to those spacer lengths for which the whole of CRN 2 was enumerated.

We found that when both the spacers were single-stranded DNA, then both toehold binding and strand displacement reactions were always plausible, irrespective of the choice of domain lengths for the spacers (figure 6*a*). Thus, CRN 2 was enumerated in all cases. This is as expected because, in our biophysical model, single-stranded spacers are assumed to be highly flexible, and when toehold binding happens, the internal diffusion takes place and a bulge is formed which always allows strand displacement to take place. When one of the spacers was changed to double-stranded, then the toehold binding reaction was always feasible, but strand displacement reaction was not plausible for the case when the length of the single-stranded spacer in nucleotides was more than twice that of the double-stranded spacer (figure 6*b*,*c*). Again, this is as expected in that the flexible single-stranded spacer must be long enough to reach past the fixed-length double-stranded spacer for the strand displacement reaction to occur. As above, the factor of two here arises from the fact that we assume the length in nanometres per nucleotide to be 0.68 nm for single-stranded DNA but only 0.34 nm for double-stranded DNA. Therefore, CRN 2 was enumerated only for the cases when double-stranded spacer is twice the length of single-stranded spacer.

Finally, when both of the spacers were double-stranded, strand displacement reactions were only plausible when both the spacer domain lengths were exactly the same (figure 6*d*). Toehold binding was plausible in all the cases; thus, CRN 1 was enumerated in all cases when the spacer lengths were different, and CRN 2 was enumerated when they were the same. Again, this is as expected because our model allowed for no flexibility of double-stranded domains, and the only way for the product structure of the strand displacement step to be geometrically plausible in this case is for the two spacers to be the same length and lined up precisely with each other. (Experimentally, it has been found that the strand displacement rate was considerably reduced when one or both spacer domains are double-stranded and that controlling spacer rigidity could thus be used to activate or deactivate strand displacement [25].) Thus, the geometric property of our system was able to capture the interplay of nature of spacers and their lengths on the dynamics of the strand displacement reaction, without any additional special-case semantic rules. We note, however, that this work only determines whether a reaction is feasible or not; as discussed below, it will be interesting to try to use similar approaches to estimate the rates of the reactions.

## 7. Related work

Representing molecules using domains, which are contiguous nucleotide sequences assumed to interact (bind and unbind) as a collective can increase the efficiency of compilation and simulations by providing an abstraction that allows us to focus on higher level designs without worrying about details at lower levels. In early work, Nishikawa *et al.* used ‘abstract bases’, a concept similar to domains, to develop the virtual nucleic acid (VNA) simulator [30]. The Reif group also developed a physically based simulation of DNA [31] and more recently published a similar graph-based approach to representing secondary structures and associated reactions [32]. Coarse-grained models such as oxDNA [33] provide a far more detailed insight into the behaviour of systems at the single-nucleotide level, at the cost of significant simulation time.

While reaction enumerators are powerful and useful in and of themselves, their utility is greatly amplified by tight integration with other tools to enable an integrated workflow. While the DyNAMiC Workbench [34] was one of the earliest attempts in this direction, one of the most complete toolkits has been assembled in recent years by the Winfree group. Of that work, the most relevant here is Peppercorn [24]. Peppercorn is a domain-based reaction enumerator that deliberately focuses on non-pseudoknotted structures, which means that molecular geometry need not be considered to enumerate reactions between species in this class. Given this restriction on the representable set of structures, a graph-based formalism is not required and Peppercorn thus uses a dotparen-style notation called ‘kernel notation’ for domain-level secondary structures, which uses parentheses to represent bound domains. For example, the representation of the unbound hairpin structure from our example in figure 2*a* would be: x(s1 y* s2). The semantics of the Peppercorn enumerator is defined via a set of rule patterns that can be matched against structures in kernel notation to determine possible reactions in a given system. Peppercorn integrates powerful support for time-scale separation and condensation of CRN models based on this time-scale separation, producing reaction networks between ‘resting states’, which are subgraphs of the network containing only fast time-scale reactions. Peppercorn also provides an approximate rate model for domain level reactions of DNA molecules, and this elegant formalism provides a more principled treatment of time-scale separation than previous work on reaction enumerators [16].

Importantly, the Peppercorn enumerator is part of an integrated toolchain developed by the Winfree group that also includes the Nuskell CRN-to-DSD compiler [35], which can convert abstract CRN specifications into DNA strand displacement implementations via several distinct encoding schemes and also verify the correctness of the encoding, the Multistrand simulator [36,37], which simulates stochastic trajectories of secondary structure dynamics at the single-base level, and the KinDA tool [38], which uses Peppercorn in conjunction with Multistrand and NUPACK [39] to determine the extent to which specific assignments of sequences to domains behave as per the assumptions inherent in domain-level design. These tools are thus perhaps the most powerful and well-integrated set of design tools for DNA strand displacement circuit design. Also of note is Piperine [6], a design platform that is specialized to the design of systems expressed as abstract CRNs using a four-domain encoding into DNA strand displacement [40] and which also links to low-level sequence design tools to enable more rapid experimental implementation of candidate designs.

The broad aim of our work is to develop reaction enumeration algorithms that are geometrically aware and can thus be applied to molecular computing systems in which geometry plays a key role. Our system can thus be generalized to enumerate a large variety of reactions, given the flexbility of the underlying strand graph data structure. Some early DNA strand displacement implementations of logic circuits [3] used metastable fuels for signal restoration reactions [41] that relied on a geometric constraint to prevent spurious output release. Perhaps the most direct use of geometry in molecular computing has been in localized circuits, in which components are tethered to an underlying surface such as a DNA origami tile [21,26], as well as molecular robotics systems [42–44]. While we do not model tethers directly in this paper, we have modelled them in previous work using satisfiability modulo theories (SMT) solving to check satisfiability of the associated geometric constraints [28], and we anticipate this will be a significant direction for future research in this area. Finally, other intriguing uses of geometry hypothesized in molecular computation include using rigid linkages to actually carry out computation via mechanical action [45].

## 8. Discussion

A key aspect of this work was to simplify the reaction rules compared with previous work on reaction enumeration [22]. We included a test for geometric plausibility, which incorporates geometric constraints into the system, and which can be applied uniformly to all candidate reactions. This approach avoids the potential for corner cases to arise from unintended consequences of the previous rules by tackling the question of geometry directly rather than attempting to approximate it via syntactic predicates on the strand graph structure, which could lead to reactions being incorrectly ruled out or in.

For example, in figure 5, we enumerated possible reactions for different domain lengths and found that, when the spacer domains were long enough, they could accommodate the binding of a single-stranded domain within the loop. To our knowledge, no previous work has considered a similar approach to enumerating binding reactions within hairpin loops, and this could therefore enable more realistic enumeration of DNA strand displacement reaction network approaches. In figure 5, we manually modified the domain lengths to determine the region of the parameter space in which the reaction could occur; however, such functionality could be automated to enable users to easily *compute* the domain lengths for which a system functions as intended. Furthermore, in reality partial binding into the loop region would exert a force on the base-pairs near to the top of the stem and open them, possibly enabling further binding into the loop, and so on. In this situation, the domain-based representation might fail to capture some details of the experimental reality. This is less an issue with our enumeration system and more an inherent weakness of the domain-based enumeration approach. A future iteration of this work might be able to model such phenomena using a more sophisticated kinetic model of DNA, which might also incorporate other enhancements to the biophysics such as an explicit treatment of the width of the double helix and the helical twist.

Our primary goal in this work was to demonstrate the integration of geometric constraints into a reaction enumeration system. Therefore, the rules of the system itself are relatively simple and we used a semantics similar to the ‘Detailed’ reaction semantics of Visual DSD [16]. This semantics does not do any merging of fast reactions, which means that some examples might not be conveniently enumerated in our prototype system due to the corresponding blow-up in the number of enumerated species. However, this would be largely due to the time-scale separation issue and not due to our geometric enumeration approach. The issue of reaction merging is orthogonal to the question of geometrically aware reaction enumeration that we study here and such a system could be added onto a geometric reaction enumerator such as ours, e.g. using an approach based on ‘resting states’, such as that used in the Peppercorn reaction enumerator [24]. Similarly, relaxing the sameSpecies constraint on the three-way branch migration rule would incorporate leak reactions, though we do not address leaks here; this could be done in future work.

We used a randomized structure sampling algorithm to try and find a satisfying set of coordinates for each structure, building upon our previous work on using geometric sampling to estimate localized reaction rates [27]. This approach has the benefit of simplicity and is applicable to any input structure given a reasonable sampling heuristic, which is a key design criterion: given that we wish to integrate our solver into the inner loop of our reaction enumeration algorithm, it must be able to run without any additional user input apart from the input structure. The efficiency of the sampling-based approach outlined here will depend in part on the number of distinct points in the structure that could lead to unsatisfiable constraints. These typically involve loops, so if a structure contains multiple independent hairpin loops then the sampler would need to sample each one independently and find a satisfying valuation for all of them in one sampling run. The likelihood of this succeeding should decrease with the number of separate hairpins. However, we have carried out some simple tests on a ‘kissing loop’ structure, where two hairpins bind via complementary sequences within the loops. Interestingly, we found that our sampling algorithm was able to find satisfying configurations for this structure quite easily (see figure S6 in the electronic supplementary material).

Furthermore, we carried out similar analysis on several pseudoknotted structures, including run times as above, and found that checking plausibility of multi-loop structures does require more samplings but seems feasible, at least for moderately complex examples (see figures S8 and S9 in the electronic supplementary material). Hence, the problem of multiple loops in input structures may not be an insurmountable problem in practical examples. Nevertheless, future work on sampling-based approaches could include efficiency optimizations such as partitioning the strand graph into independent sub-graphs, sampling each sub-graph separately, then combining these solutions in an attempt to produce a solution for the entire strand graph. Further work might also be to give stronger guarantees of non-plausibility in the case that our sampling algorithm fails. Indeed, the results of an initial random sampling could be used as a starting point for further numerical optimization, if required.

The advantage of our general-purpose geometric framework for reaction enumeration is that other approaches to solving geometric constraints could be used instead. For example, in previous work, we used SMT solving to check for satisfiability of geometric constraints: while exact SMT solvers for nonlinear constraints over the reals do exist [46], their worst-case time complexity meant that run times proved intractable for some examples that could not easily be predicted in advance, which was not practical. Therefore, our earlier work actually used a less exact solver based on floating-point representations [28]. An alternative approach would be direct solving of the geometric constraints based on numerical optimization techniques such as distance matrix completion [47], although the results of these can be sensitive to the initial guesses for the parameters, meaning that multiple attempts might be required, like the sampling-based approach employed here. Another possibility would be to try to adapt exact constraint solvers developed for the fields of mainstream robotics and motion planning [48]. There are intriguing crossovers between those fields and the geometric analysis of DNA nanostructures, although the tools from one field may not be directly applicable to the other: we anticipate that attempting to apply existing software tools from robotics to biomolecular computing would be an interesting direction for future research.

In the reaction rules above, every rule application checks for geometric plausibility. In a more sophisticated implementation, we could skip some of these checks if certain rules can be proven to preserve plausibility and the initial species fed into the system are plausible. For example, the ‘unbind’ rule should preserve plausibility since the only change is the removal of a bond, which only serves to *remove* some constraints. We might also be able to further optimize the system by reintroducing special case rules in the interest of efficiency, for example, in the case of a three-way branch migration reaction when the next domain is directly adjacent to the displacement domain. Thus, the geometric constraint system would give a concrete mathematical justification for the special-case rules.

Finally, our geometric approach to reaction enumeration lends itself very well to model localized components in a tethered reaction system. We anticipate that an extension of the system presented here could be used to automatically generate kinetic models of tethered reaction systems based on DNA strand displacement [21,26], automatically taking geometry into account to determine which reactions between tethered components are actually possible in practice. A further challenge would be to calculate reasonable estimates of the local concentrations (and thus reaction rates) for intramolecular reactions, if we want to model the kinetics of these automatically, building on our previous work [27]. The work presented here could therefore be a powerful tool for the modelling of localized molecular circuits, which are a promising tool for the future development of fast and scalable molecular computing systems.

## Data Availability

Data is accessible via the electronic supplementary material. Code is available under an open source license from Github: https://github.com/matthewlakin/GeometricEnumerator/. Supplementary material is available online [49].

## Footnotes

1 https://github.com/matthewlakin/GeometricEnumerator/.

## References

[1] Zhang DY, Winfree E. 2009 Control of DNA strand displacement kinetics using toehold exchange. J. Am. Chem. Soc. **131**, 17 303-17 314. (10.1021/ja906987s) 19894722

[2] Zhang DY. 2011 Cooperative hybridization of oligonucleotides. J. Am. Chem. Soc. **133**, 1077-1086. (10.1021/ja109089q) 21166410

[3] Seelig G, Soloveichik D, Zhang DY, Winfree E. 2006 Enzyme-free nucleic acid logic circuits. Science **314**, 1585-1588. (10.1126/science.1132493) 17158324

[4] Qian L, Winfree E. 2011 Scaling up digital circuit computation with DNA strand displacement cascades. Science **332**, 1196-1201. (10.1126/science.1200520) 21636773

[5] Chen YJ, Dalchau N, Srinivas N, Phillips A, Cardelli L, Soloveichik D, Seelig G. 2013 Programmable chemical controllers made from DNA. Nat. Nanotechnol. **8**, 755-762. (10.1038/nnano.2013.189) 24077029 PMC4150546

[6] Srinivas N, Parkin J, Seelig G, Winfree E, Soloveichik D. 2017 Enzyme-free nucleic acid dynamical systems. Science **358**, eaal2052. (10.1126/science.aal2052) 29242317

[7] Allen PB, Arshad SA, Li B, Chen X, Ellington AD. 2012 DNA circuits as amplifiers for the detection of nucleic acids on a paperfluidic platform. Lab. Chip **12**, 2951-2958. (10.1039/c2lc40373k) 22729075 PMC3454488

[8] Hemphill J, Deiters A. 2013 DNA computation in mammalian cells: microRNA logic operations. J. Am. Chem. Soc. **135**, 10 512-10 518. (10.1021/ja404350s) 23795550

[9] Lopez R, Wang R, Seelig G. 2018 A molecular multi-gene classifier for disease diagnostics. Nat. Chem. **10**, 746-754. (10.1038/s41557-018-0056-1) 29713032

[10] Jung C, Ellington AD. 2014 Diagnostic applications of nucleic acid circuits. Acc. Chem. Res. **47**, 1825-1835. (10.1021/ar500059c) 24828239 PMC4063332

[11] Chen YJ, Groves B, Muscat RA, Seelig G. 2015 DNA nanotechnology from the test tube to the cell. Nat. Nanotechnol. **10**, 748-760. (10.1038/nnano.2015.195) 26329111

[12] Li S et al. 2018 A DNA nanorobot functions as a cancer therapeutic in response to a molecular trigger *in vivo*. Nat. Biotechnol. **36**, 258-264. (10.1038/nbt.4071) 29431737

[13] Cherry KM, Qian L. 2018 Scaling up molecular pattern recognition with DNA-based winner-take-all neural networks. Nature **559**, 370-376. (10.1038/s41586-018-0289-6) 29973727

[14] Zarubiieva I, Spaccasassi C, Kulkarni V, Phillips A. 2022 Automated leak analysis of nucleic acid circuits. ACS Synth. Biol. **11**, 1931-1948. (10.1021/acssynbio.2c00084) 35544754

[15] Phillips A, Cardelli L. 2009 A programming language for composable DNA circuits. J. R. Soc. Interface **6**, S419-S436. (10.1098/rsif.2009.0072.focus) 19535415 PMC2843959

[16] Lakin MR, Youssef S, Cardelli L, Phillips A. 2012 Abstractions for DNA circuit design. J. R. Soc. Interface **9**, 460-486. (10.1098/rsif.2011.0343) PMC3262419 21775321

[17] Lakin MR, Youssef S, Polo F, Emmott S, Phillips A. 2011 Visual DSD: a design and analysis tool for DNA strand displacement systems. Bioinformatics **27**, 3211-3213. (10.1093/bioinformatics/btr543) 21984756 PMC3208393

[18] Lakin MR, Phillips A. 2011 Modelling, simulating and verifying Turing-powerful strand displacement systems. In *Proc. of the 17th Int. Conf. on DNA Computing and Molecular Programming* (eds L Cardelli, W Shih), vol. 6937, *Lecture Notes in Computer Science*, pp. 130–144. New York, NY: Springer. (10.1007/978-3-642-23638-9_12)

[19] Dalchau N, Chandran H, Gopalkrishnan N, Phillips A, Reif J. 2015 Probabilistic analysis of localized DNA hybridization circuits. ACS Synth. Biol. **4**, 898-913. (10.1021/acssynbio.5b00044) 26133087

[20] Lakin MR, Petersen R, Gray KE, Phillips A. 2014 Abstract modelling of tethered DNA circuits. In *Proc. of the 20th Int. Conf. on DNA Computing and Molecular Programming* (eds S Murata, S Kobayashi), vol. 8727, *Lecture Notes in Computer Science*, pp. 132–147. Cham, Switzerland: Springer International Publishing. (10.1007/978-3-319-11295-4_9)

[21] Chatterjee G, Dalchau N, Muscat RA, Phillips A, Seelig G. 2017 A spatially localized architecture for fast and modular DNA computing. Nat. Nanotechnol. **12**, 920-927. (10.1038/nnano.2017.127) 28737747

[22] Petersen RL, Lakin MR, Phillips A. 2016 A strand graph semantics for DNA-based computation. Theor. Comput. Sci. **632**, 43-73. (10.1016/j.tcs.2015.07.041) 27293306 PMC4896506

[23] Spaccasassi C, Lakin MR, Phillips A. 2019 A logic programming language for computational nucleic acid devices. ACS Synth. Biol. **8**, 1530-1547. (10.1021/acssynbio.8b00229) 30372611

[24] Badelt S, Grun C, Sarma KV, Wolfe B, Shin SW, Winfree E. 2020 A domain-level DNA strand displacement reaction enumerator allowing arbitrary non-pseudoknotted secondary structures. J. R. Soc. Interface **17**, 20190866. (10.1098/rsif.2019.0866) 32486951 PMC7328391

[25] Genot AJ, Zhang DY, Bath J, Turberfield AJ. 2011 Remote toehold: a mechanism for flexible control of DNA hybridization kinetics. J. Am. Chem. Soc. **133**, 2177-2182. (10.1021/ja1073239) 21268641

[26] Bui H, Shah S, Mokhtar R, Song T, Garg S, Reif J. 2018 Localized DNA hybridization chain reactions on DNA origami. ACS Nano **12**, 1146-1155. (10.1021/acsnano.7b06699) 29357217

[27] Kumar S, Weisburd JM, Lakin MR. 2021 Structure sampling for computational estimation of localized DNA interaction rates. Sci. Rep. **11**, 12730. (10.1038/s41598-021-92145-8) 34135406 PMC8209221

[28] Lakin MR, Phillips A. 2018 Automated analysis of tethered DNA nanostructures using constraint solving. Nat. Comput. **17**, 709-722. (10.1007/s11047-018-9693-y)

[29] Thirumalai D, Ha BY. 1998 Statistical mechanics of stiff chains. In *Theoretical and mathematical methods in polymer research* (ed. AY Grosberg), pp. 1–35. New York, NY: Academic Press.

[30] Nishikawa A, Yamamura M, Hagiya M. 2001 DNA computation simulator based on abstract bases. Soft Comput. **5**, 25-38. (10.1007/s005000000062)

[31] Sahu S, Wang B, Reif JH. 2008 A framework for modeling DNA based molecular systems. J. Comput. Theor. Nanosci. **5**, 2124-2134. (10.1166/jctn.2008.1108)

[32] Mokhtar R, Garg S, Chandran H, Bui H, Song T, Reif J. 2017 Modeling DNA nanodevices using graph rewrite systems. In *Advances in unconventional computing volume 2: prototypes, models and algorithms* (ed. A Adamatzky), Emergence, Complexity, and Computation, vol. 23, pp. 347–395. Cham, Switzerland: Springer International Publishing.

[33] Doye JPK et al. 2013 Coarse-graining DNA for simulations of DNA nanotechnology. Phys. Chem. Chem. Phys. **15**, 20 395-20 414. (10.1039/C3CP53545B) 24121860

[34] Grun C, Werfel J, Zhang DY, Yin P. 2015 DyNAMiC Workbench: an integrated development environment for dynamic DNA nanotechnology. J. R. Soc. Interface **12**, 20150580. (10.1098/rsif.2015.0580) 26423437 PMC4614494

[35] Badelt S, Shin SW, Johnson RF, Dong Q, Thachuk C, Winfree E. 2017 A general-purpose CRN-to-DSD compiler with formal verification, optimization, and simulation capabilities. In *Proc. of the 23rd Int. Conf. on DNA Computing and Molecular Programming* (eds R Brijder, L Qian), vol. 10467, *Lecture Notes in Computer Science*, pp. 232–248. Cham, Switzerland: Springer International Publishing. (10.1007/978-3-319-66799-7_15)

[36] Schaeffer JM, Thachuk C, Winfree E. 2015 Stochastic simulation of the kinetics of multiple interacting nucleic acid strands. In *Proc. of the 21st Int. Conf. on DNA Computing and Molecular Programming* (eds A Phillips, P Yin), vol. 9211, *Lecture Notes in Computer Science*, pp. 194–211. Cham, Switzerland: Springer International Publishing. (10.1007/978-3-319-21999-8_13)

[37] Srinivas N, Ouldridge TE, Šulc P, Schaeffer JM, Yurke B, Louis AA, Doye JPK, Winfree E. 2013 On the biophysics and kinetics of toehold-mediated DNA strand displacement. Nucleic Acids Res. **41**, 10 641-10 658. (10.1093/nar/gkt801) PMC3905871 24019238

[38] Berleant J, Berlind C, Badelt S, Dannenberg F, Schaeffer J, Winfree E. 2018 Automated sequence-level analysis of kinetics and thermodynamics for domain-level DNA strand-displacement systems. J. R. Soc. Interface **15**, 20180107. (10.1098/rsif.2018.0107) 30958232 PMC6303802

[39] Zadeh JN, Steenberg CD, Bois JS, Wolfe BR, Pierce MB, Khan AR, Dirks RM, Pierce NA. 2011 NUPACK: analysis and design of nucleic acid systems. J. Comput. Chem. **32**, 170-173. (10.1002/jcc.21596) 20645303

[40] Soloveichik D, Seelig G, Winfree E. 2010 DNA as a universal substrate for chemical kinetics. Proc. Natl Acad. Sci. USA **107**, 5393-5398. (10.1073/pnas.0909380107) 20203007 PMC2851759

[41] Seelig G, Yurke B, Winfree E. 2006 Catalyzed relaxation of a metastable DNA fuel. J. Am. Chem. Soc. **128**, 12 211-12 220. (10.1021/ja0635635) 16967972

[42] Yin P, Yan H, Daniell XG, Turberfield AJ, Reif JH. 2004 A unidirectional DNA walker that moves autonomously along a track. Angew. Chem. Int. Ed. **43**, 4906-4911. (10.1002/anie.200460522) 15372637

[43] Gu H, Chao J, Xiao SJ, Seeman NC. 2010 A proximity-based programmable DNA nanoscale assembly line. Nature **465**, 202-205. (10.1038/nature09026) 20463734 PMC2872101

[44] Aarnio M, Sankila R, Pukkala E, Salovaara R, Aaltonen LA, Peltomäki P, Mecklin JP, Järvinen HJ. 1999 Cancer risk in mutation carriers of DNA-mismatch-repair genes. Int. J. Cancer **81**, 214-218. (10.1002/(SICI)1097-0215(19990412)81:2<214::AID-IJC8>3.0.CO;2-L) 10188721

[45] Breik K, Luchsinger A, Soloveichik D. 2021 Molecular machines from topological linkages. In *27th Int. Conf. on DNA Computing and Molecular Programming (DNA 27)* (eds MR Lakin, P Šulc), vol. 205, *Leibniz International Proceedings in Informatics (LIPIcs)*, pp. 7:1–7:20. Schloss Dagstuhl – Leibniz-Zentrum für Informatik.

[46] Jovanović D, de Moura L. 2012 Solving non-linear arithmetic. In *Proceedings of IJCAR 2012*, vol. 7364, *LNCS*, pp. 339–354. Berlin, Germany: Springer. (10.1007/978-3-642-31365-3_27)

[47] Trosset MW. 2000 Distance matrix completion by numerical optimization. Comput. Optim. Appl. **17**, 11-22. (10.1023/A:1008722907820)

[48] Somani N, Rickert M, Knoll A. 2017 An exact solver for geometric constraints with inequalities. IEEE Rob. Autom. Lett. **2**, 1148-1155. (10.1109/LRA.2017.2655113)

[49] Kumar S, Lakin MR. 2023 A geometric framework for reaction enumeration in computational nucleic acid devices. Figshare. (10.6084/m9.figshare.c.6916113) PMC10645505 37963554

